# Kidney involvement in children during the SARS-CoV-2 Omicron variant pandemic

**DOI:** 10.1186/s12887-023-04322-5

**Published:** 2023-09-28

**Authors:** Jiwon Jung, Jina Lee, Joo Hoon Lee

**Affiliations:** Department of Pediatrics, Asan Medical Center Children’s Hospital, University of Ulsan College of Medicine, 88, Olympic-Ro 43-Gil, Songpa-Gu, Seoul, 05505 Republic of Korea

**Keywords:** SARS-CoV-2 variant infection, Children, Nephrotic syndrome, Hematuria, Proteinuria

## Abstract

**Background:**

As the coronavirus disease-2019 (COVID-19) pandemic continues, driven by the Omicron variant, infection rates in children have recently rapidly surged compared with previous years. We aimed to investigate the presentation of kidney involvement in children after Omicron variant severe acute respiratory syndrome coronavirus-2 (SARS-CoV-2) infection.

**Methods:**

We retrospectively reviewed the medical records of pediatric patients who presented with kidney disease with a temporal relationship with COVID-19 between January and August 2022 in a single tertiary center in Korea.

**Results:**

Fifteen children presented with kidney involvement after Omicron variant infection, with a median age of 10.6 (6.8–18.3) years. None of the patients exhibited severe respiratory symptoms apart from cough and sore throat. The median time from infection to renal symptom onset was 3 (0–49) days. Among 10 patients with underlying kidney disease, six had previously been diagnosed with nephrotic syndrome (NS) that relapsed after COVID-19 infection, two with immunoglobulin A nephropathy (IgAN) experienced transient gross hematuria (GHU) with or without acute kidney injury (AKI), and two with kidney transplantation presented with AKI. Of the five patients without underlying kidney disease, one patient had NS, and the other four patients had GHU and proteinuria (PU), of whom one was eventually diagnosed with Henoch Shönlein Purpura nephritis (HSPN), and one with rhabdomyolysis. The seven patients with NS (1 new-onset, 6 relapsed) had uneventful remission with corticosteroid therapy. Apart from one patient with new-onset HSPN, GHU and PU resolved spontaneously in all affected patients, and AKI also resolved with supportive care.

**Conclusions:**

Kidney involvement subsequent to Omicron variant COVID-19 exhibited various, but mostly mild manifestations in children.

## Introduction

As the COVID-19 pandemic persisted with initial fatal variants, it became evident that the impact of COVID-19 affects not only the respiratory system. Major organs such as the heart, brain, liver, kidney, as well as vascular and endocrine systems, were also affected [1, 2]. Kidney damage stands among the recognized consequences of COVID-19, attributed to both direct invasion of podocytes and proximal tubular cells, as well as indirect effects such as immune dysregulation, endothelial dysfunction, and hypercoagulability [2]. Various kidney pathologies have been reported with this viral infection across all age groups, including collapsing glomerulopathy, acute tubular necrosis, IgA nephropathy (IgAN), thrombotic microangiopathy, crescentic glomerulonephritis, minimal change disease, membranous nephropathy, non-collapsing focal segmental glomerulosclerosis (FSGS), and anti-glomerular basement membrane (GBM) disease [3].

At the beginning of the pandemic, children were considered to be at lower risk of COVID-19, with a lower incidence of infection and milder clinical course, even among those with chronic immunosuppressant use for conditions such as kidney disease [4–6]. However, sporadic reports of kidney involvement in COVID-19 in children have emerged. Although large-scale epidemiological studies and comprehensive pathology reviews specifically focusing on pediatric patients with COVID-19 are lacking, small cohort studies and case reports have highlighted kidney involvement between 2020 and 2021. These studies predominantly highlighted kidney involvement, mainly with the wild type, Alpha, and Delta variant-related SARS-CoV-2 infections. Bjornstad et al. reported a high prevalence (44%) of acute kidney injury (AKI) among critically ill children with COVID-19 between April and May 2020, which is comparable with the finding among critically ill adult patients [7]. Wu et al. conducted a meta-analysis of children with kidney involvement after COVID-19 infection and reported cases of new-onset and relapsed nephrotic syndrome (NS), glomerulonephritis, and atypical hemolytic uremic syndrome up to October 31, 2021 [8].

**Fig. 1:**
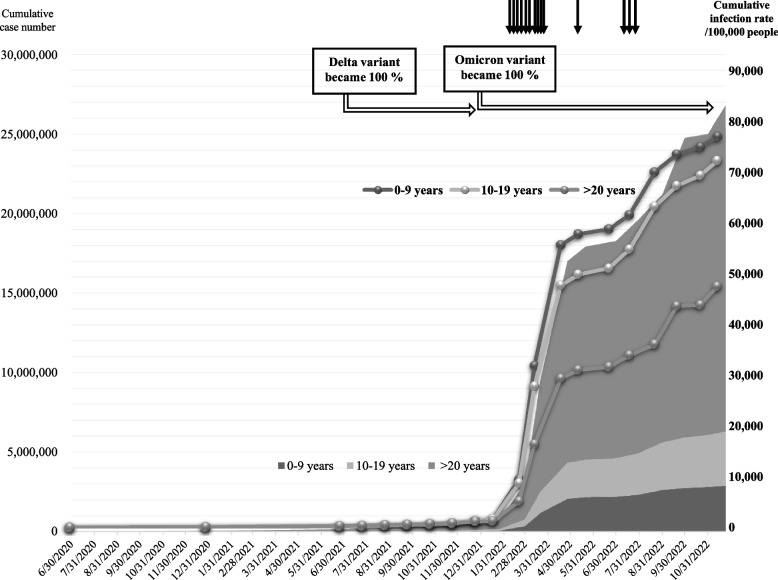
Cumulative cases and rate of COVID-19 infection in Korea according to age groups. By the end of January 2022, the Omicron variant had become the predominant strain, accounting for nearly 100% of cases in South Korea. Subsequently, an explosive increase in confirmed cases has occurred since 2022, especially in children. The cumulative number of confirmed COVID-19 cases in Korea is shown for different age groups, represented by varying shades, corresponding to the values on the left axis. The curved line graphs depict the cumulative incidence rate of cases per 100,000 populations in Korea, corresponding to the values on the right axis. The age group of 0–9 years exhibited the highest incidence rate, followed by those aged 10–19 years. The empty arrows indicate the period when each dominant variant emerged. The arrows at the top represent each case, based on their time of presentation in this study

As the COVID-19 pandemic endured, the Delta variant emerged as the predominant strain, accounting for nearly 100% of all detected SARS-CoV-2 strains by August 2021. Subsequently, by the end of January 2022, the Omicron variant gained prevalence, exhibiting a nearly 100% detection rate in South Korea [9–11]. Given the high contagious nature of the Omicron variant and the implementation of government’s quarantine mitigation policy, the number of confirmed cases, particularly among children, witnessed an exponential increase, accounting for 20% of all confirmed cases across all ages groups in South Korea (Fig. 1) [10]. Despite the unprecedented surge in Omicron variant SARS-CoV-2 infections among children, both within South Korea and worldwide, only a limited number of reports have delved into Omicron-associated kidney involvement patterns. Consequently, we aimed to report Omicron variant SARS-CoV-2 infection cases with kidney from a single tertiary center and evaluate the severity of kidney involvement in children.

## Methods

We included pediatric patients who visited the Asan Medical Center Children’s Hospital owing to kidney involvement after contracting COVID-19 between January and August 2022. The temporal relationship was defined as kidney involvement occurring within 2 months of COVID-19 infection and not explained by other causes. We retrospectively collected medical records encompassing pertinent cases, including demographics, post- COVID-19 symptoms, kidney involvement characteristics, management approaches, and outcomes. COVID-19 was confirmed via positive real-time (RT)- polymerase chain reaction (PCR), and concordant symptoms with positive rapid antigen tests performed by medical staff were also considered valid, particularly considering Omicron prevalence. Patients diagnosed with COVID-19 during this period were assumed to have the Omicron variant infection, given its dominance post-December 2021 and the absence of additional noteworthy variants of interest up to August 2022.

Continuous variables are presented as median (range), and the Mann–Whitney *U* test was performed to compare the median duration of kidney involvement after COVID-19 between patients with underlying kidney disease and those without. A *p*-value of < 0.05 was considered statistically significant. The estimated glomerular filtration rate (eGFR) was calculated using the bedside Chronic Kidney Disease in Children study (CKiD) formula [12]. COVID-19 was classified as mild (confirmed infection by RT-PCR not necessitating hospitalization), moderate (hospitalization required without intensive care unit [ICU] admission, mechanical ventilation, or death), and severe/critical (ICU admission, mechanical ventilation, or death) based on the classification in previous literature [13].

## Results

Table 1 present a summary of the clinical characteristics and outcomes for the 15 patients who exhibited kidney involvement following COVID-19 infection during the Omicron variant pandemic. None of the patients displayed severe respiratory symptoms necessitating respiratory support. The patients experienced fever that subsided within 3 days except for patient 14, who had a persistent fever lasting 8 days. Patient 14 also presented with upper respiratory symptoms (URS), such as cough and sore throat, along with myalgia. Among the cohort, 12 (80.0%) had mild COVID-19, whereas three (20.0%) had moderate disease requiring admission for kidney related issues such as AKI or rhabdomyolysis, rather than for respiratory symptoms.

**Table 1:** Characteristics and clinical features of patients with kidney involvement after COVID-19 infection

Pt	Age	Sex	Kidney	COVID-19		Intervals	Underlying	Medication	Bx	Management	Outcome
No	(y)		involvement	Severity	Vaccination	(d)	disease	at presentation			
1^a^	9.5	F	New-onset NS	Mild	-	42	-	-	-	Steroid	remission in 7d
2	11.2	M	NS relapse, GHU	Mild	-	3	SDNS (3.0y-)	FK506/ACEi	-	Steroid	remission in 10d
3	6.8	M	NS relapse	Mild	-	23	SDNS (3.2y-)	-	-	Steroid	remission in 14d
4	9.7	F	NS relapse	Mild	-	21	SSNS (4.9y-)	-	-	Steroid	remission in 6d
5	4.8	F	NS relapse	Mild	-	7	SSNS (3.9y-)	-	-	Steroid	remission in 9d
6	8.8	M	NS relapse	Mild	-	1	SRNS (3.5y-,)	FK506/steroid	MCD	Steroid	remission in 9d
7	12.0	F	NS relapse	Mild	-	1	SRNS (3.4y-)	-	MCD	Steroid	remission in 8d
8	7.5	M	GHU/PU (PrCR0.5)	Mild	-	0	KD (3y)	-	-	Observe	PU: Resolved in 9d
							HSP (6y)				mHU: persist
9	15.0	M	GHU/PU (PrCR1.4)	Moderate	-	2	CD (9.8y-)	Adalimumab	-	Hydration	RML/PU: Improved
			RML								mHU: persistent
10	12.9	F	GHU/PU (PrCR0.3)	Mild	-	0	-	-	-	Observe	mHU/PU: persist
11	12.8	F	GHU/AKI (eGFR 66 ml/min/m^2^)	Mild	1	6	IgAN (9.5y-)	ARB	IgAN	Observe	improved in 41d
12	14.1	M	GHU	Mild	2	0	IgAN (14.0y-)	AZA/ACEi	IgAN	Observe	mHU: persistent
13^a^	9.1	F	GHU/NS (PrCR14.5)	Mild	-	49	DBA (birth-)	-	HSPN^a^	Steroid,	PU: Resolved in 47d
			after HSP							AZA	mHU: persistent
14	10.6	M	AKI (eGFR 73 ml/min/m^2^)	Moderate	-	0	KT (5.8y, ARPKD)	FK506/MMF	-	Hydration	resolution in 2d
15	18.3	M	AKI (eGFR 49 ml/min/m^2^)	Moderate	3	18	KT (17.9y, IgAN)	FK506/MMF/steroid	-	Hydration	resolution in 112d

^a^Newly diagnosed case after COVID-19 infection

*Abbreviations*: *Pt* Patient, *No.* Number, *y* Years, *d* Days, *F* Female, *M* Male, *NS* Nephrotic syndrome, *GHU* Gross hematuria, *PU* Proteinuria, *PrCR* Urine protein/creatinine ratio, *SDNS* Steroid-dependent nephrotic syndrome, *SSNS* Steroid-sensitive nephrotic syndrome *SRNS* steroid-resistant nephrotic syndrome, *Bx* Kidney biopsy finding, *MCD* Minimal change disease, *IgAN* Immunoglobulin A nephropathy, *KD* Kawasaki disease, *HSP* Henoch-Shönlein purpura, *CD* Crohn's disease, *DBA* Diamond-Blackfan anemia 1, *AKI* Acute kidney injury, *eGFR* Estimated glomerular filtration rate, *KT* Kidney transplantation, *ARPKD* Autosomal recessive polycystic kidney disease, *FK506* Tacrolimus, *ACEi* Angiotensin converting enzyme inhibitor, *ARB* Angiotensin receptor blocker, *AZA* Azathioprine, *MMF* Mycophenolate mofetil

Ten (66.7%) patients had underlying kidney disease, among which six patients (60.0%, patient 2, 3, 4, 5, 6, and 7) with previously diagnosed NS experienced a relapse after COVID-19. Two patients with IgAN (20.0%, patient 11, 12) had transient gross hematuria (GHU) with or without AKI. Additionally, two patients (20.0%, patient 14, 15) who had undergone kidney transplantation developed transient AKI following diarrhea or dehydration with severe pharyngeal ulceration. Patient 15, the latter case with pharyngeal ulcer, experienced persistent fever for 7 days, leading to a 5-day course of Remdesivir administration. The fever subsided within 2 days, resulting in patient discharge. However, 1 week post-discharge, the patient presented with AKI coupled with a tacrolimus blood level 3 times above normal.

Five (33.3%) patients without a previous history of kidney disease presented with new-onset kidney involvement. Among them, one patient presented with NS (patient 1). Four patients (80.0%, patient 8, 9, 10, and 13) exhibited GHU and proteinuria (PU), with one of them also experiencing concomitant rhabdomyolysis (patient 9), while another had Henoch Shönlein Purpura (HSP) (patient 13).

The median age of the study participants was 10.6 (range 6.8–18.3) years, and the male-to-female ratio was 8:7. The median duration from COVID-19 confirmation to the onset of kidney involvement symptoms was 3 days (range, 0–49). Regarding patients with underlying kidney disease, the median duration from SARS-CoV-2 infection to the onset of kidney involvement symptom was 4.5 days (range 0–21), a difference not found to be statistically significant compared with those without underlying kidney disease (2 days [range 0–49]; *p* = 0.901). Two patients had long intervals between COVID-19 and kidney involvement (patient 1, and 13). Patient 1 showed persistent URS for > 1 month, and developed progressive leg edema 3 weeks after SARS-CoV-2 infection. She was diagnosed with new onset NS when she presented at the nephrology clinic at 42 days after infection. Patient 13 presented with pruritic purpura across both lower extremities, as well as edema, and purpura on the trunk and arms 18 days after COVID-19, which was accompanied by a single day of fever, sore throat, and myalgia. Given the ongoing presence of purpura 49 days after COVID-19 and identification of anomalies in urinalysis, including nephrotic-range proteinuria (urine protein/creatinine ratio (PrCR) 23.46 mg/mg), microscopic hematuria (mHU) (RBC Many/HPF), hypoalbuminemia (2.8 g/dL), and hypogammaglobulinemia (371.6 mg/dL), along with normal kidney function, with a connection to COVID-19 could not be discounted. Kidney biopsy showed diffuse mesangial proliferative glomerulonephritis, consistent with HSP nephritis (Hass classification subclass IV, Oxford classification M1 E1 S0 T0 C0). The patient achieved remission of PU in 47 days with deflazacort followed by mycophenolate mofetil, but had persistent mHU (RBC 21–30/HPF) and normal kidney function (eGFR 95–110 ml/min/m^2^) for the subsequent 8 months.

Of the 15 study patients, a mere three (20.0%) had received COVID-19 vaccination prior to experiencing kidney involvement. Patient 15 had successfully completed all three vaccine doses, patient 12 had received two doses, and patient 1 had received solely the first dose, however, subsequent doses were withheld owing to the occurrence of GHU and hypotension following vaccination. The remaining 12 (80.0%) patients had not received any vaccination at the juncture of their COVID-19 diagnosis.

## Discussion

In this case series, we have delineated the features of kidney involvement in children following the widespread surge of Omicron variant SARS-CoV-2 infection within a Korean tertiary center. The scope of kidney involvement encompassed various presentations, including new onset NS, or NS relapse, transient GHU coupled with proteinuria, new-onset HSPN, rhabdomyolysis, and transient renal insufficiency in patients with established kidney conditions, including those with prior transplants. Remarkably all cases responded favorably to standard treatment or exhibited self–limiting course, without progression to sustained renal insufficiency.

Within this study, patients presented with symptoms that primarily included fever, myalgia, and URS. However, no patient displayed symptoms necessitating oxygen therapy, lower respiratory tract symptoms, respiratory failure, critical illness, or multi-organ involvement, regardless of the presence of underlying kidney disease. These observations align with the characteristics typically associated with Omicron variant infection, which generally manifest with a much milder trajectory than wild-type or Delta variant infections. This trend holds true, even among children < 5 who lack vaccination or previous infection, corroborating findings from reports concerning adult infections [13–15].

Although the majority of patients in this study demonstrated mild disease in association with the Omicron variant, moderate disease occurred in three out of six patients with underlying diseases that required an immunomodulatory drug (adalimumab in patient 9 with Crohn’s disease, and calcineurin inhibitor combined with mycophenolate in 2 kidney transplantation patients, patient 14, and patient 15). Patients being administered tacrolimus (for NS in patients 2 and 6) or azathioprine (for IgAN in patient 12) alone exhibited mild COVID-19. The variance in disease severity might stem from differences in the intensity and duration of immunosuppression. A notable Italian cohort study focused on pediatric and adolescent patients with NS undergoing chronic immunosuppressive therapy (anti-CD20) during the early COVID-19 pandemic (February–July 2020). Initially, this study suggested that chronic immunosuppression did not increase the risk of COVID-19 in children, even in a region with a high COVID-19 prevalence [4]. However, the same group subsequently reported a rapid increase (0%–13%) of COVID-19 infections among patients undergoing chronic immunosuppressive therapy (21% using Rituximab and 8% using mycophenolate mofetil, *p* = 0.03) during the Omicron variant pandemic. Despite these infections, fatalities did not ensue [16], findings that parallel the outcomes observed in our study.

The NS emerged as the prevailing diagnosis in cases of Omicron-associated kidney disease, including new-onset NS, NS relapse with uneventful remission following steroid therapy was the most frequently encountered scenario in our study. Throughout the study period, we observed only one Omicron-associated new-onset NS, compared with three patients who developed new-onset NS without Omicron infection. Several instances of new-onset NS during the early stages of the wild-type COVID-19 pandemic were reported [17, 18]. When contrasting our case with instances of new onset NS cases documented in the previous literature during the infection incubation period, a distinct pattern emerged. Specifically, patient 1, our case, was received a diagnosis of NS 42 days after COVID-19 confirmation. This prolonged interval was coupled with the presence of persistent URS that endured for > 1 month, alongside progressive leg edema. Notably, this constellation of symptoms raised suspicions of a potential “post-COVID-19 condition” [19]. Because SARS-CoV-2 can be a trigger for NS relapse following upper respiratory tract infection [20, 21], several reports have revealed relapse after pre-Omicron variant COVID-19 infection. However, the number of cases was not higher than that before the pandemic, and, these cases responded well to standard steroid treatment [22, 23]. Although estimating and comparing the exact incidence is challenging because the study was not designed to establish a NS patient cohort, 6 out of 135 patients with NS (4.4%) who visited the hospital during the study period experienced relapse. When compared with the same period before the COVID-19 pandemic (NS relapse occurred in 7 out of 120 patients (5.8%) between January and August 2019), the relapse rate was seemingly not increased during the Omicron pandemic. This infrequent relapse was also shown in an Italian NS cohort with a 34.7% (61/176) Omicron variant infection rate, among whom only 1 patient relapsed (1/176, 0.5%)[24]. Another single-center Korean study showed an Omicron-associated relapse rate of 15% (3/20), which was comparable with the relapse rate of patients with NS without Omicron variant infection (20.5%, 8/39) during the same period (February–June 2022). These cases showed a favorable treatment response [25]. These findings might be attributed in part to a lower incidence of viral infections other than COVID-19 owing to mask-wearing and distancing measure implemented during the pandemic.

This study had no cases of aggressive progressive glomerulopathy, such as acute necrotizing glomerulopathy, collapsing glomerulopathy, or atypical hemolytic uremic syndrome. In contrast, studies among adults indicated that AKI with or without multi-organ failure was observed during the early stages of the COVID-19 pandemic [26]. Self-limiting transient GHU or IgAN (HSPN) was the feature of Omicron-associated glomerulopathy from our center. Compared with the two cases of new-onset HSPN without Omicron variant infection during the same period, only one case of Omicron-associated HSPN occurred. There was no case requiring advanced kidney replacement therapy such as dialysis. Although a case of Delta variant-related HSPN in a 5-year-old Caucasian boy was previously documented before September 2021 [27], our report on the patient with HSP, who had mild abnormalities on urinalysis (patient 13) is the first report on Omicron-associated HSPN. This case displayed a positive response to standard management without renal insufficiency.

Prior to the prevalence of the Omicron variant, reports regarding COVID-19-related rhabdomyolysis in children were absent. A Taiwanese group recently reported eight cases of rhabdomyolysis during 2 months of the Omicron epidemic (from April–June 2022) in a centralized quarantine center [28]. None of these patients had underlying diseases, and seven out of the eight patients were not vaccinated for COVID-19. After the fever caused by Omicron SARS-CoV-2 infection resolved (in 3–5 days), rhabdomyolysis was diagnosed based on severe calf pain, CK levels ranging from 1346–6937 U/L, and abnormal urinary findings. This differs from the presentation of our patient 9, who had an underlying disease (being administered adalimumab), exhibited GHU at presentation, and experienced no calf pain. All patients, including patient 9, had excellent outcomes with supportive care, indicating a benign and self-limiting course.

Regarding children who had undergone kidney transplantation, Varnell et al. reported a low incidence of COVID-19 (0.6%, 10/1686) among the Improving Renal Outcomes Collaborative (IROC) cohort in the early days of the COVID-19 pandemic (April–September 2020) [29]. No severe cases were observed, and all cases showed excellent short-term outcomes without mortality, respiratory failure, or allograft loss. Only three patients required temporal reduction in the mycophenolate mofetil dose. This contrasted with adult cases, where individuals who had undergone kidney transplantation and were on immunosuppressive therapy experienced unfavorable graft outcomes after COVID-19 [30, 31]. Nonetheless, in adult kidney transplantation patients, the overall COVID-19-related mortality decreased after the prevalence of the Omicron variant compared with that in the earlier phase of the pandemic [32]. Based on these findings, a better outcome could be expected in pediatric transplantation patients after an Omicron infection. AKI in patient 14 and 15 may be partly attributed to pre-renal etiology because there was severe diarrhea and dehydration exacerbated by poor oral intake. Furthermore, although unlikely, an interaction between immunosuppressive agents and COVID-19 medications might have contributed, as seen in patient 15 who was administered Remdesivir. This medication is a cytochrome P450 34A inhibitor, and could potentially interact with other drugs, including tacrolimus, and Paxlovid (nirmatrelvir combined with ritonavir), leading to increased plasma levels [33, 34]. Although there were no instances of new onset nephropathy or renal insufficiency requiring kidney replacement therapy, it is important to remain vigilant and closely monitor the possibility of both clinical and subclinical AKI after infection among transplantation patients.

Most of the patients in our study had not been vaccinated against SARS-CoV-2 before their infection. Only one patient (patient 15) received three vaccine doses, another (patient 12) had received two doses, and remaining patients discontinued further vaccination after the first dose after experiencing adverse effects such as hypotension and GHU. COVID-19 vaccination was approved for children aged 12–17 and 5–11 on October 18, 2021, and February 23, 2022, respectively, in Korea. However, because the incidence of Omicron variant infections had surged among Korean children by that time, the vaccination completion rate among children remained low owing to parental reluctance after natural infection. Had the patients acquired immunity against COVID-19 through full vaccination before infection, the spectrum and severity of COVID-19 symptoms may have differed. Therefore, it is essential for medical personnel to educate and encourage patients and their caregivers on the importance of vaccination and provide sufficient information regarding its benefits, and possible complications.

This study has some limitations. Being a case series from a single center in Korea, its findings cannot be generalized as a representative feature of kidney involvement after COVID-19. However, our case series may be meaningful in the absence of accumulated reports of kidney involvement after the rapid increase in the incidence of COVID-19 among children. Additionally, we could not calculate the exact incidence of kidney involvement among patients with COVID-19. The exact count of SARS-CoV-2-infected children remained uncertain as COVID-19 cases were identified either through the patient/guardian reports outside our center or direct RT-PCR testing at our center. Furthermore, subclinical kidney involvement, such as mHU or mild AKI, could have gone unnoticed as they might not have exhibited noticeable symptoms. Finally, kidney biopsy was only performed for patient 13 owing to the favorable outcomes seen in most patients, without clinical indications of progressive nephropathy. Despite these limitations, this study remains valuable as it outlines the characteristics of relatively benign kidney involvement after Omicron SARS-CoV-2 infection in children, during a time when cases of children with COVID-19 began to surge for the first time since 2019.

## Conclusion

In conclusion, kidney involvement, including HU, PU, or AKI, can occur infrequently in children after Omicron variant SARS-CoV-2 infection, leading to favorable outcomes. Even with a milder course of infection, vigilance is crucial, as various forms of kidney involvement with or without pre-existing kidney conditions may require careful monitoring among children.

## Data Availability

Patient data cannot be fully accessed due to local research ethics protocols. Specific data may be available from the corresponding author.

## Abbreviations

AKI: Acute kidney injury
COVID-19: Corona virus disease-2019
eGFR: Estimated glomerular filtration rate
FSGS: Focal segmental glomerulosclerosis
GBM: Glomerular basement membrane
GH: Gross hematuria
HSP: Henoch Shönlein Purpura
ICU: Intensive care unit
IgAN: IgA nephropathy
mHU: Microscopic hematuria
NS: Nephrotic syndrome
PU: Proteinuria
RT-PCR: Real-time-polymerase chain reaction
URS: Upper respiratory symptoms
SARS-CoV-2: Severe acute respiratory syndrome coronavirus-2
